# D139N mutation of PsbP enhances the oxygen-evolving activity of photosystem II through stabilized binding of a chloride ion

**DOI:** 10.1093/pnasnexus/pgac136

**Published:** 2022-07-23

**Authors:** Ko Imaizumi, Taishi Nishimura, Ryo Nagao, Keisuke Saito, Takeshi Nakano, Hiroshi Ishikita, Takumi Noguchi, Kentaro Ifuku

**Affiliations:** Division of Integrated Life Science, Graduate School of Biostudies, Kyoto University, Kyoto 606-8502, Japan; Division of Integrated Life Science, Graduate School of Biostudies, Kyoto University, Kyoto 606-8502, Japan; Division of Material Science, Graduate School of Science, Nagoya University, Nagoya 464-8602, Japan; Research Institute for Interdisciplinary Science, Okayama University, Okayama 700-8530, Japan; Research Center for Advanced Science and Technology, The University of Tokyo, Tokyo 153-8904, Japan; Department of Applied Chemistry, The University of Tokyo, Tokyo 113-8654 , Japan; Division of Integrated Life Science, Graduate School of Biostudies, Kyoto University, Kyoto 606-8502, Japan; Research Center for Advanced Science and Technology, The University of Tokyo, Tokyo 153-8904, Japan; Department of Applied Chemistry, The University of Tokyo, Tokyo 113-8654 , Japan; Division of Material Science, Graduate School of Science, Nagoya University, Nagoya 464-8602, Japan; Division of Applied Life Sciences, Graduate School of Agriculture, Kyoto University, Kyoto 606-8502, Japan

**Keywords:** photosynthesis, oxygen evolution, chloride ions, membrane-extrinsic proteins

## Abstract

Photosystem II (PSII) is a multisubunit membrane protein complex that catalyzes light-driven oxidation of water to molecular oxygen. The chloride ion (Cl^−^) has long been known as an essential cofactor for oxygen evolution by PSII, and two Cl^−^ ions (Cl-1 and Cl-2) have been found to specifically bind near the Mn_4_CaO_5_ cluster within the oxygen-evolving center (OEC). However, despite intensive studies on these Cl^−^ ions, little is known about the function of Cl-2, the Cl^−^ ion that is associated with the backbone nitrogens of D1-Asn338, D1-Phe339, and CP43-Glu354. In green plant PSII, the membrane extrinsic subunits—PsbP and PsbQ—are responsible for Cl^−^ retention within the OEC. The Loop 4 region of PsbP, consisting of highly conserved residues Thr135–Gly142, is inserted close to Cl-2, but its importance has not been examined to date. Here, we investigated the importance of PsbP-Loop 4 using spinach PSII membranes reconstituted with spinach PsbP proteins harboring mutations in this region. Mutations in PsbP-Loop 4 had remarkable effects on the rate of oxygen evolution by PSII. Moreover, we found that a specific mutation, PsbP-D139N, significantly enhances the oxygen-evolving activity in the absence of PsbQ, but not significantly in its presence. The D139N mutation increased the Cl^−^ retention ability of PsbP and induced a unique structural change in the OEC, as indicated by light-induced Fourier transform infrared (FTIR) difference spectroscopy and theoretical calculations. Our findings provide insight into the functional significance of Cl-2 in the water-oxidizing reaction of PSII.

Significance Statement Photosystem II (PSII) catalyzes the oxidation of water to molecular oxygen. Chloride ions are indispensable for this reaction, and the PsbP and PsbQ subunits play a crucial role in their retention in the green plant PSII. We examined the importance of the Loop 4 region of PsbP, located near Cl-2, one of the two chloride ions bound in the oxygen-evolving center of PSII. Mutations in PsbP-Loop 4 significantly affect the oxygen-evolving activity of PSII. A unique mutation, PsbP-D139N, markedly enhances this activity in the absence of PsbQ. This mutation stabilizes the binding of Cl-2 to PSII by inducing a specific structural change around the Mn_4_CaO_5_ cluster. Our work suggests the importance of Cl-2 in the water-oxidizing reaction of PSII.

## Introduction

Oxygenic photosynthesis converts light energy into chemical energy and produces oxygen from water, sustaining aerobic life forms on Earth. Photosystem II (PSII), a pigment–protein complex found in oxygenic photosynthetic organisms, initiates photosynthesis by catalyzing the light-driven water-oxidizing reaction (1). The catalytic center of the water-oxidizing reaction is the oxygen-evolving center (OEC), which includes the inorganic Mn_4_CaO_5_ cluster organized in a “distorted chair” form (2, 3). In total, two water molecules are converted into one molecular oxygen, four protons, and four electrons in a stepwise manner through the S-state cycle (S*_i_; i* = 0 to 4) of the OEC, where S_0_ and S_4_ are the most reduced and oxidized states, respectively (4). S_1_ is the most dark-stable state, and each S*_i_* (*i* = 0 to 3) state is advanced to the next S*_i_*_+1_ state by flash illumination. Release of molecular oxygen occurs during the final step S_3_ (→ S_4_) → S_0_, after the transient S_4_ state.

**Fig. 1.**
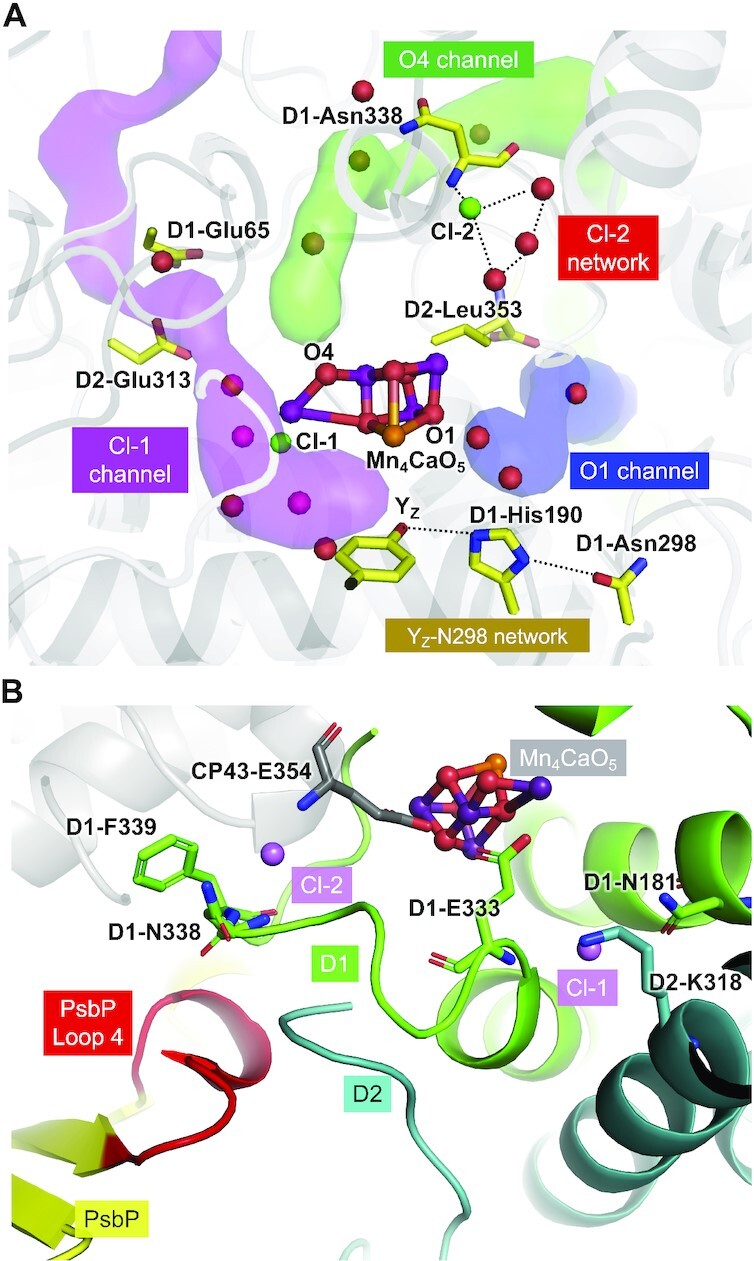
Structures of water channels, hydrogen-bond networks, Cl^−^ binding sites, and PsbP-Loop 4 in the OEC of green plant PSII. (A) Structures of the O4, O1, and Cl-1 (E65/E312; E65/E313 in pea) channels and the Cl-2 and Y_Z_-N298 networks in PSII from pea (PDB ID: 5XNL). The channel space was analyzed using Caver (108). The O4 channel was analyzed in spinach PSII and it is composed of D1, D2, CP43, and PsbP subunits (6). The components of the O4 channel in the pea PSII are identical to those in the spinach PSII. (B) The Cl^−^ binding sites and the location of the Loop 4 region of PsbP (PDB ID: 5XNL). D1, D2, and PsbP are shown in green, blue, and yellow cartoon view, respectively, and the Loop 4 region of PsbP is colored in red. The Cl^−^ ions are shown as pink spheres. The amino acid residues associated to the Cl^−^ ions are shown as stick models.

Studies on cyanobacterial PSII have provided detailed information on the structure of the OEC, and this has been found to be similar in green plant PSII (2, 5–7). The Mn_4_CaO_5_ cluster is surrounded by several hydrogen-bonded water channels and networks, which could be involved in the exit of protons or the inlet of water molecules (2, 8–14) (Fig.1A). Additionally, two chloride ions (Cl^−^), Cl-1 and Cl-2, bind in the vicinity of the Mn_4_CaO_5_ cluster; Cl-1 is the Cl^−^ ion whose binding site is near the amino group of D2-Lys317, the side chain of D1-Asn181, and the backbone nitrogen of D1-Glu333, whereas Cl-2 binds adjacent to the backbone nitrogens of D1-Asn338, D1-Phe339, and CP43-Glu354 (2, 15, 16) (Fig.1B). The Cl-1 binding site is estimated to have a higher Cl^−^ binding affinity compared to the Cl-2 binding site (17). The water channels and hydrogen-bond networks found around the Mn_4_CaO_5_ cluster in cyanobacterial PSII are as follows: the Cl-1 channel (also known as the “E65/E312 channel” (18) or the “broad channel” (9)) (2, 19–21), the O1-channel (also known as the “large channel” (9)) (6, 8, 18, 22), the Y_Z_-N298 network (2, 12, 23, 24), the O4-channel (also known as the “narrow channel” (9)) (18, 22, 25–28), and the Cl-2 network (18). As shown in Fig.1(A), similar water channels and hydrogen-bond networks are observed in green plant PSII (6).

Cl^−^ ions are known to be required for the oxygen-evolving activity of PSII, but the functions of the Cl^−^ ions had long been a subject of debate (29–35). However, recent studies, most of which use cyanobacterial PSII, have advanced our understandings, especially of the functions of Cl-1 (36). D1-Glu333 and CP43-Glu354, involved in binding of Cl-1 and Cl-2, respectively, are both directly coordinated to the Mn_4_CaO_5_ cluster (2). Therefore, the Cl^−^ ions have been proposed to affect the conformational structures of the OEC (2), and this has been supported for Cl-1 based on EPR studies and theoretical studies (37–45). Additionally, Cl-1 has been indicated to be important for the hydrogen-bond network within the Cl-1 channel (16, 17, 46), which is suggested to be a proton transfer pathway (20, 21). The theoretical study by Mandal et al. (47) further revealed the contribution of Cl-1 to the oxygen-evolving activity of PSII. On the other hand, very few studies have focused on Cl-2, as the hydrogen-bond network proceeding from the Mn_4_CaO_5_ cluster through the Cl-2 binding site does not reach the bulk surface (18). Therefore, the functions of Cl-2 and whether or not Cl-2 is important for the oxygen-evolving activity of PSII are yet to be elucidated.

PSII is composed of a number of membrane-intrinsic proteins and several membrane-extrinsic proteins (48). The extrinsic subunits surround the Mn_4_CaO_5_ cluster to form the OEC, and they are involved in both Cl^−^ retention and the hydrogen-bond networks (49). However, while the basic subunit structure of the PSII core is highly conserved among the diverse oxyphototrophs, a drastic evolutionary change is observed in the composition of the extrinsic subunits (49–52). Green plants, including land plants and green algae, have a set of three extrinsic subunits, PsbO, PsbP, and PsbQ, which bind to the lumenal side of PSII (53), while cyanobacterial PSII binds PsbU and PsbV instead of PsbP and PsbQ (54, 55). PsbU and PsbV are also present in red algal and diatom PSII, and structural comparison of the PSII complexes from cyanobacteria, red algae, diatoms, and green plants show that PsbP in green plant PSII spatially replaces PsbU and PsbV in the other types of PSII (5, 36, 49). Recent studies have also shown that water channels involving extrinsic subunits in cyanobacterial PSII (O4-PsbU channel and O1-PsbU/PsbV channel) are structurally conserved as PsbP-related water channels in green plant PSII (O4-PsbP channel and O1-PsbP channel) (6).

Both in vitro and in vivo experiments have revealed the molecular functions of PsbP and PsbQ in previous reports. In vitro reconstitution experiments have shown that PsbP is important for the retention of the Cl^−^ and Ca^2+^ ions, which are indispensable for the oxygen-evolving activity of PSII (56–58). FTIR analyses have suggested that PsbP plays a role in inducing conformational changes in the OEC, especially around the Cl^−^ ion(s) (59, 60). A recent high-speed atomic force microscopy (HS-AFM) study has also suggested the importance of PsbP together with PsbO for the OEC structure (61). In vivo analyses using PsbP knockout and knockdown plants have revealed the essential roles of PsbP in photoautotrophy and assembly of PSII complexes (62–65). PsbQ, on the other hand, supported Cl^−^ retention only in reconstitution experiments performed under low Cl^−^ conditions (58, 66, 67), and was not essential for photoautotrophic growth in plants (62). Therefore, PsbP is particularly important for optimizing the Cl^−^ and Ca^2+^ availability and enhancing the oxygen-evolution reaction, while PsbQ may play an auxiliary role in supporting the functions of PsbP.

Recent cryo-electron microscopy (cryo-EM) structures of green plant PSII–Light-harvesting complex II (LHCII) supercomplexes (5, 7) have shown that a loop region of PsbP (Loop 4, consisting of the residues from Thr135 to Gly142) is inserted in close proximity to the C-terminus of D2 and the C-terminal loop region of D1, located near Cl-2 (Fig.1B). This C-terminal region of D1 interacts with both the Mn_4_CaO_5_ cluster and Cl-2, and the C-terminus of D2 is in its vicinity. In this study, we investigated the role of PsbP-Loop 4 near Cl-2 using mutated PsbP proteins. We found a novel mutation, PsbP-D139N, enhancing the oxygen-evolving activity of PSII. This PsbP-D139N mutation increased the Cl^−^ retention ability of PsbP, and its effect on the protein conformational change in the OEC was suggested by FTIR analysis and theoretical calculation. Our results give insight into the functional significance of Cl-2 for the oxygen-evolving reaction.

## Results

### PsbP-Loop 4 affects the oxygen-evolving activity of PSII

**Fig. 2.**
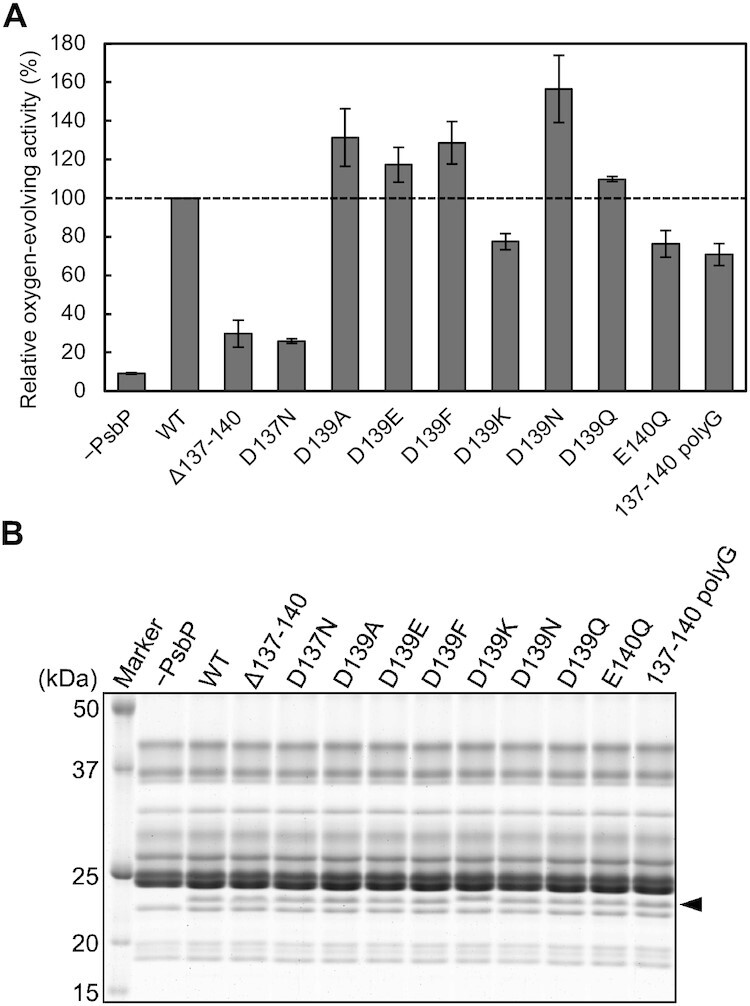
Effects of various mutations in PsbP-Loop 4 on the oxygen-evolving activity of PSII. (A) The oxygen-evolving activities of PSII membranes reconstituted with various PsbP mutant proteins were measured in buffer (25 mM MES-NaOH, 0.4 M sucrose, pH 6.5) with 0.4 mM DCBQ as an electron acceptor. The sample “−PsbP” is NaCl-washed PSII without reconstitution of PsbP. Oxygen-evolving activity of WT-reconstituted PSII (177 to 190 µmol O_2_ mg Chl^−1^ h^−1^ in independent experiments) was set as 100%; error bars = SD (*n* = 3, technical replicates). (B) PSII membranes reconstituted with various PsbP mutant proteins were subjected to SDS-PAGE in order to confirm the binding of the recombinant PsbPs. Proteins equivalent to 3 µg chlorophyll were loaded onto each lane, and the gels were stained with Oriole stain (Bio-Rad). The arrow head indicates PsbP-6xHis bands.

The amino acid sequence of the Loop 4 region of PsbP is highly conserved among green plants (Figure S1 and Table S1, Supplementary Material). To investigate the importance of the PsbP-Loop 4 region, we constructed recombinant PsbP proteins with or without mutations in the Loop 4 region (Wild-type [WT], D137N, D139A, D139E, D139F, D139K, D139N, D139Q, E140Q, and Δ137–140, where the four residues from Asp137 to Glu140 were deleted, and 137-140 polyG, where all three of Asp137, Asp139, and Glu140 were replaced with Gly). The 6xHis-tags, consisting of six consecutive histidine residues, were attached to the C-terminus of these PsbP proteins. Deciding from the cryo-EM structures of green plant PSII (5, 7, 68), the C-terminal His-tag should not affect the oxygen-evolving activity of PSII, as was the case with PSII in *Chlamydomonas reinhardtii* (69). The recombinant PsbP proteins were reconstituted to NaCl-washed PSII membranes, which are depleted of only PsbP and PsbQ, and their oxygen-evolving activity was measured in a buffer without Cl^−^ and Ca^2+^ (Fig.2A; Table S2, Supplementary Material). Subsequently, the PsbP-reconstituted PSII membranes were subjected to SDS-PAGE to confirm the binding of PsbP to PSII (Fig.2B). In order to examine the effects of mutations in PsbP-Loop 4, PsbQ was not reconstituted here, as previous reports have shown that the presence of PsbQ can mask the effects of mutations in PsbP (66, 70). Mutations in PsbP-Loop 4 showed remarkable effects on the oxygen-evolving activity of PSII, with little effect on the extent of PsbP binding to PSII. Among the 10 mutations examined, D137N and ∆137–140 led to the lowest oxygen-evolving activity, with only 20% to 30% of that of PSII reconstituted with WT PsbP. In contrast, D139N-reconstituted PSII revealed a significantly increased oxygen-evolving activity of approximately 150% relative to that of WT-reconstituted PSII. The effects of other substitution mutations at Asp139 varied depending on the amino acid residue replacing it. While substitution of Asp139 with electrically neutral or acidic amino acids resulted in a relatively high oxygen-evolving activity, D139K-reconstituted PSII showed a reduction in the activity. E140Q- and 137-140 polyG-reconstituted PSII also exhibited a decrease in oxygen-evolving activity to 70% to 80% of that of WT-reconstituted PSII. These results clearly show that the Loop 4 region of PsbP plays an important role in optimizing the oxygen-evolving activity of PSII.

**Fig. 3.**
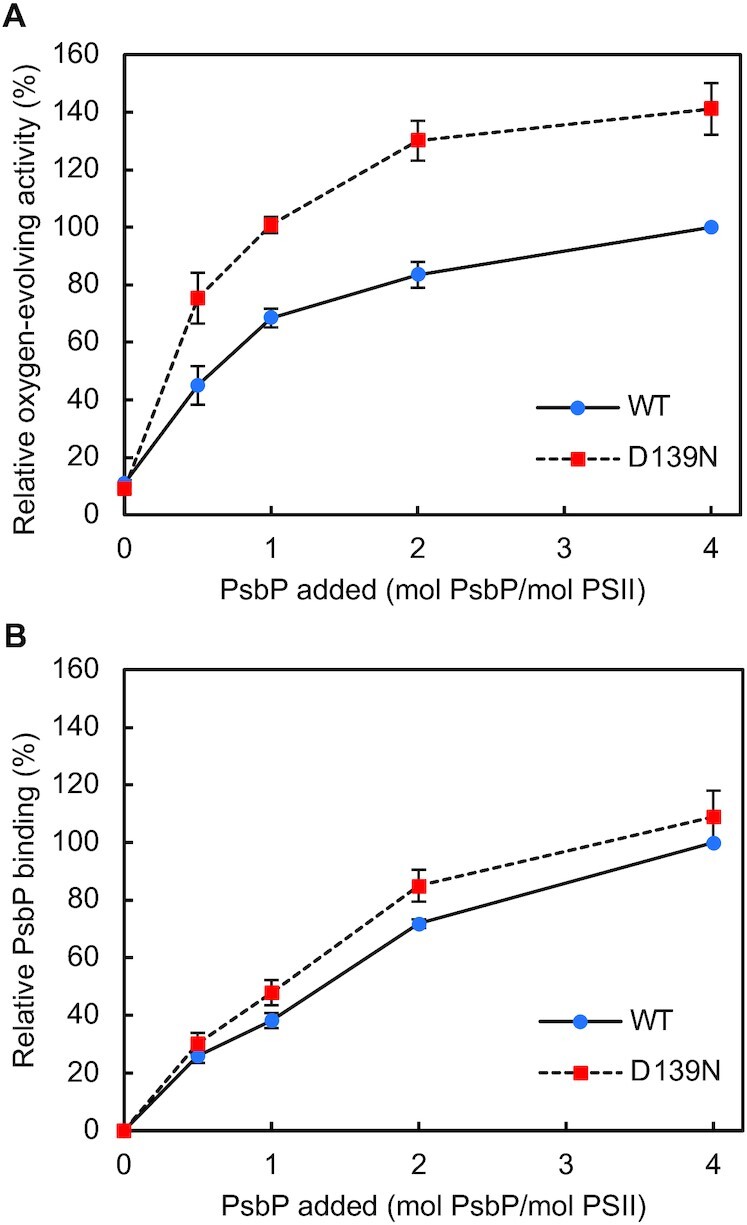
Effect of the PsbP-D139N mutation on the oxygen-evolving activity of PSII and binding of PsbP to PSII. PSII membranes were reconstituted with various amounts of PsbP (WT (blue circles) or D139N (red squares)), and (A) the oxygen-evolving activity and (B) the extent of PsbP binding was measured. For (A), oxygen-evolving activity of PSII reconstituted with WT PsbP at a ratio of PsbP: PSII = 4:1 (157 to 167 µmol O_2_ mg Chl^−1^ h^−1^ in independent experiments) was set as 100%; error bars = SD (*n* = 3, technical replicates), and for (B), the amount of WT PsbP bound to PSII when reconstituted with a ratio of PsbP: PSII = 4:1 was set as 100%; error bars = SD (*n* = 3, technical replicates).

For a more precise comparison of the binding ability of the WT PsbP protein and the D139N mutant PsbP protein to PSII, WT PsbP proteins and D139N mutant PsbP proteins were reconstituted to NaCl-washed PSII membranes with various PsbP: PSII ratios, and oxygen-evolving activity measurements were conducted (Fig. 3A), followed by SDS-PAGE analysis and quantification of the PsbP bands (Fig. 3B; Figure S2, Supplementary Material). While the oxygen-evolving activity of D139N-reconstituted PSII was higher than that of WT-reconstituted PSII, there was little difference in the binding ability of the WT and D139N mutant PsbP proteins. The curves obtained for the oxygen-evolving activity and the curves obtained for PsbP-binding were similar between WT- and D139N-reconstituted PSII, indicating that the specific binding of PsbP to PSII was not affected by the D139N mutation.

### PsbP-D139N mutation enhances the Cl^−^ retention ability of PsbP

**Fig. 4.**
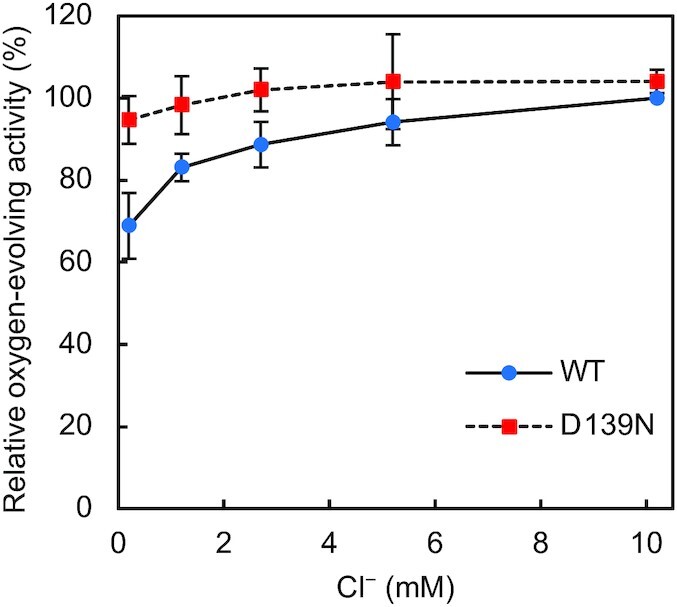
Cl^−^ dependence of the oxygen-evolving activity of WT- and D139N-reconstituted PSII. The oxygen-evolving activity of WT- (blue circles) and D139N- (red squares) reconstituted PSII was measured in the presence of various concentrations of Cl^−^ at pH 6.5. Oxygen-evolving activity of WT-reconstituted PSII under 10.2 mM Cl^−^ (241 to 282 µmol O_2_ mg Chl^−1^ h^−1^ in independent experiments) was set as 100%; error bars = SD (*n* = 3, technical replicates).

Further analyses were conducted on the PsbP-D139N mutation, which exceptionally enhanced the oxygen-evolving activity of PSII, to clarify the mechanisms underlying this enhancement. Considering that PsbP plays a crucial role in retaining Cl^−^ and Ca^2+^, and that the Loop 4 region of PsbP is located near Cl-2, we determined whether the D139N mutation of PsbP affects the Cl^−^ dependence of the oxygen-evolving activity of PSII (Fig. 4). After reconstitution of WT PsbP and D139N mutant PsbP proteins to PSII under sufficient Cl^−^, the oxygen-evolving activity was assayed under various Cl^−^ concentrations up to approximately 10 mM, the optimum Cl^−^ concentration (58). Although the increase in oxygen-evolving activity by the PsbP-D139N mutation was observed with all Cl^−^ concentrations examined, the difference with WT-reconstituted PSII was more prominent at lower Cl^−^ concentrations. The low Cl^−^ dependence of the oxygen-evolving activity of D139N-reconstituted PSII membranes, even at extremely low Cl^−^ concentrations, suggests a higher Cl^−^ retention ability obtained by the mutation. The low Cl^−^ dependence of the oxygen-evolving activity of untreated PSII binding both WT-PsbP and PsbQ, and the high Cl^−^ dependence of that of NaCl-washed PSII has been confirmed in Figure S3 (Supplementary Material).

In the meanwhile, although PsbP is also important for Ca^2+^ retention, the PsbP-D139N mutation did not affect the Ca^2+^ dependence of the oxygen-evolving activity (Figure S4A, Supplementary Material). Furthermore, the observation that the effect of the PsbP-D139N mutation does not depend on the light intensity (Figure S4B, Supplementary Material), nor the type of electron acceptor quinones (Table S3, Supplementary Material), supports a particular effect of the mutation on the OEC. Taken together, these results suggest that the PsbP-D139N mutation specifically enhanced the retention of Cl^−^ in the OEC.

### PsbP-D139N mutation alters the pH dependence of oxygen-evolving activity

**Fig. 5.**
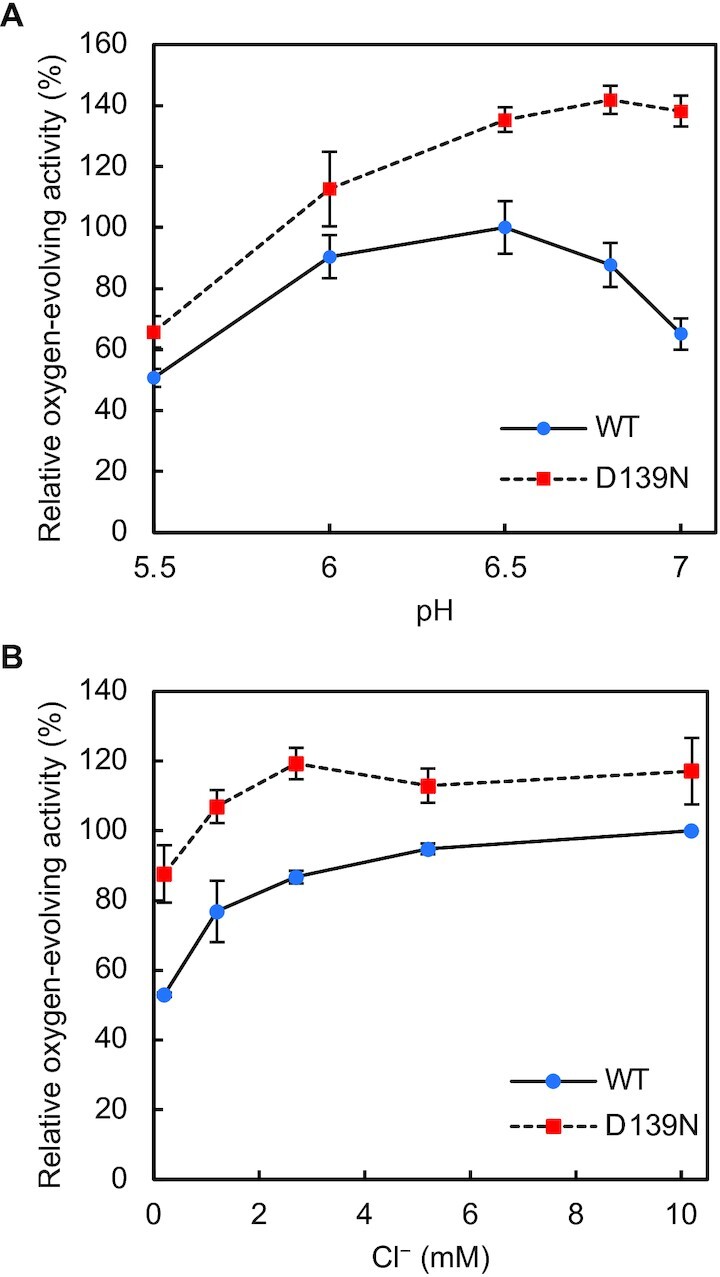
pH dependence and effect of Cl^−^ at pH 7.0 on the oxygen-evolving activity of WT- and D139N-reconstituted PSII. The oxygen-evolving activity of WT- (blue circles) and D139N- (red squares) reconstituted PSII was measured (A) at different pH (under 0.2 mM Cl^−^), (B) in the presence of various concentrations of Cl^−^ at pH 7.0. Oxygen-evolving activity of WT-reconstituted PSII (A) at pH 6.5 (142 µmol O_2_ mg Chl^−1^ h^−1^), (B) under 10.2 mM Cl^−^ (225 to 239 µmol O_2_ mg Chl^−1^ h^−1^ in independent experiments) was set as 100%; error bars = SD (*n* = 3, technical replicates).

We next examined the pH dependence of the oxygen-evolving activity of WT- and D139N-reconstituted PSII membranes in the pH range from pH 5.5 to pH 7.0 (Fig. 5A). D139N-reconstituted PSII showed a higher oxygen-evolving activity than WT-reconstituted PSII in the above pH range, and the enhancement of activity by the PsbP-D139N mutation was larger at higher pH. In both WT-reconstituted PSII and D139N-reconstituted PSII, the oxygen-evolving activity increased with the increase of pH from pH 5.5 to pH 6.5. However, while the oxygen-evolving activity of WT-reconstituted PSII dropped upon further increase of pH from the optimum pH 6.5 to pH 7.0, the activity of D139N-reconstituted PSII remained high at pH values above pH 6.5. It has been reported that the Cl^−^ dependence of the oxygen-evolving activity of PSII increases with the increase in pH (71–73). Therefore, the high activity of D139N-reconstituted PSII at higher pH values could have been caused by the enhanced Cl^−^ retention ability. In fact, the Cl^−^ dependence of the oxygen-evolving activity of WT- and D139N-reconstituted PSII examined at pH 7.0, showed that the difference between the oxygen-evolving activity of WT- and D139N-reconstituted PSII was larger under very low Cl^−^ concentrations (Fig. 5B). These data suggest that the observed alteration of the pH dependence of oxygen-evolving activity could be due to stabilized Cl^−^ binding by the D139N mutation even at elevated pH.

### PsbP-D139N mutation induces a unique conformational change around the Mn_4_CaO_5_ cluster

**Fig. 6.**
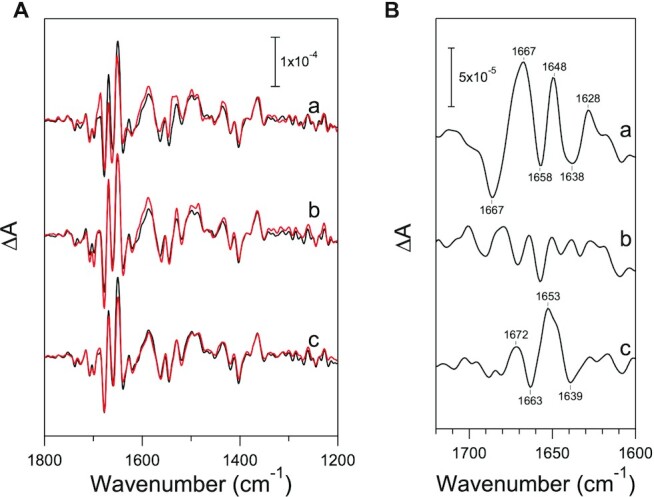
Effect of the PsbP-D139N mutation on the S_2_/S_1_ FTIR difference spectrum of PSII membranes. (A) Light-induced S_2_/S_1_ FTIR difference spectra of untreated (a–c, black lines), NaCl-washed (a, red line), WT-reconstituted (b, red line), and D139N-reconstituted (c, red line) PSII membranes. (B) The amide I region of the untreated-minus-treated double-difference spectra of the S_2_/S_1_ FTIR difference spectra: (a) untreated-minus-NaCl-washed, (b) untreated-minus-WT-reconstituted, and (c) untreated-minus-D139N-reconstituted PSII.

To further investigate the effect of the D139N mutation, light-induced Fourier transform infrared (FTIR) difference spectroscopy was used to probe the structural changes around the Mn_4_CaO_5_ cluster. Light-induced FTIR difference spectroscopy is a powerful tool that enables the detection of structural changes coupled with the stepwise water-oxidizing reaction (74), and has been used to investigate the effect of PsbP on the structures in the OEC (59, 60, 66, 75–77). The conformational change around the Mn_4_CaO_5_ cluster upon S_1_→S_2_ transition was examined by FTIR analyses of NaCl-washed, WT-reconstituted, and D139N-reconstituted PSII. The light-induced FTIR difference spectrum upon only Q_A_ reduction (Q_A_^−^/Q_A_) was subtracted from that associated with the formation of an S_2_Q_A_^−^ charge separated state (S_2_Q_A_^−^/S_1_Q_A_) to obtain the FTIR difference spectrum upon the S_1_→S_2_ transition (S_2_/S_1_; Fig. 6A). Depletion of PsbP and PsbQ induced a significant change in the spectral feature in the amide I region (1,700 to 1,600 cm^−1^), resulting in prominent peaks in the untreated-minus-treated double difference spectrum (Fig. 6B, line a), whereas they were markedly restored by reconstitution of WT PsbP (Fig. 6B, line b), consistent with observations in previous studies (59, 66, 75, 76). On the other hand, reconstitution of the D139N mutant PsbP led to amide I features, which differed from those of NaCl-washed as well as WT-reconstituted PSII (Fig. 6B, line c). The amide I bands arise from the CO stretching vibrations of backbone amides around the Mn_4_CaO_5_ cluster (59, 60, 78). Thus, the results in Fig. 6 suggest that the D139N mutant PsbP induces a conformational change, involving the movement of polypeptide backbones, different from that induced by WT PsbP upon the S_1_→S_2_ transition. Moreover, considering that the change in the amide I bands due to binding of PsbP has been attributed to conformational changes, including those around the Cl^−^ ion(s) (60), the unique structural change observed with D139N-reconstituted PSII can be speculated to have taken place around the Cl^−^ ion(s), as well.

### Structural changes induced by the PsbP-D139N mutation were investigated by theoretical calculations

**Fig. 7.**
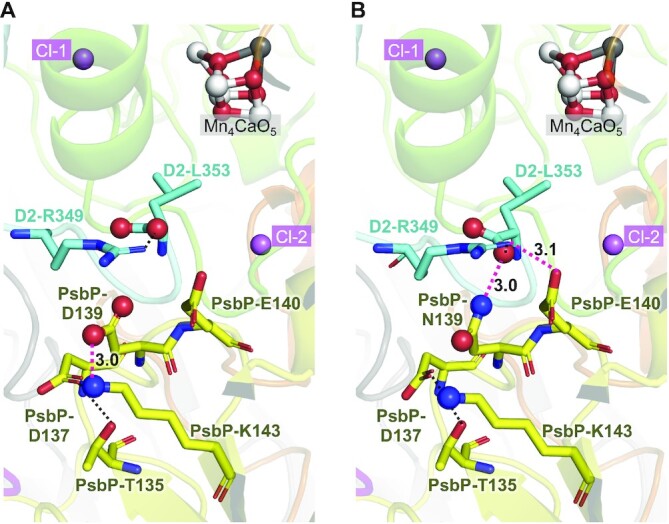
Optimized protein structures of (A) WT PSII and (B) PsbP-D139N mutant PSII obtained by calculations based on the reported PSII structure (PDB ID: 5XNL). D1, D2, and PsbP are shown in green, blue, and yellow cartoon view, respectively. Dotted lines indicate interactions (hydrogen-bonds and salt-bridges) between the side chains of amino acid residues, and those that are formed/diminished by the D139N mutation are colored in pink, with the N–O distances [Å] shown. The hydrogen atoms are not shown for clarity. In the alternative conformation where the Nδ atom of PsbP-Asn139 is oriented toward the side chain of PsbP-Lys143 (Figure S5, Supplementary Material), the calculated energy was 16.1 kcal mol^−1^ higher than that of the original conformation where the Nδ atom of PsbP-Asn139 is hydrogen-bonded with the backbone carboxyl group of D2-Leu353 as shown in (B).

To examine the effect of the D139N mutation of PsbP on the structure of PSII, a comparison of the PSII structure with and without the PsbP-D139N mutation was made based on theoretical calculations (Fig. 7; Figure S5, Supplementary Material). The PsbP-Asp139 side chain forms a salt bridge with the PsbP-Lys143 side chain in the WT PSII (3.0 Å N–O distance; Fig. 7A), whereas in the PsbP-D139N mutant PSII, the PsbP-Asn139 side chain forms a hydrogen bond with the backbone carboxyl group of D2-Leu353 (3.0 Å N–O distance; Fig. 7B). In the PsbP-D139N mutant PSII, the formation of a hydrogen bond between the PsbP-Glu140 and D2-Arg349 side chains is also observed (3.1 Å N–O distance).

## Discussion

In this study, we discovered a highly unique mutation, PsbP-D139N, that significantly increased the oxygen-evolving activity of PSII compared to WT PsbP. The Loop 4 region of PsbP is located near Cl-2 (Fig. 1B), and analyses of the PsbP-D139N mutation have suggested the importance of Cl-2 for the oxygen-evolving activity of PSII.

Mutations in the Loop 4 region of PsbP greatly affected the oxygen-evolving activity of PSII (Fig. 2A). The remarkably low activity with the Δ137–140 mutation lacking the Loop 4 region indicates the crucial role of this loop for the optimal oxygen-evolving activity. The D137N mutation also led to a dramatic decrease in oxygen-evolving activity, suggesting the importance of the PsbP-Asp137 residue. Sakashita et al. (6) previously reported that the O4-PsbU channel connecting the O4 site of the Mn_4_CaO_5_ cluster with the protein bulk surface in cyanobacterial PSII (26) is structurally conserved in green plant PSII as the O4-PsbP channel. There, it was suggested that the salt bridge between PsbP-Asp137 and CP43-Lys339 in the O4-PsbP channel of green plant PSII corresponded to that between PsbU-Asp96 and CP43-Lys339 in the O4-PsbU channel of cyanobacterial PSII, and that these conserved salt bridges had a role in the channel orientation. Therefore, the substantial decline in oxygen-evolving activity observed by the D137N mutation is expected to have been caused by the effect on the channel orientation of the O4-PsbP channel. While little is known about the proton transfer pathways in green plant PSII, this result is indicative of the functioning of the O4-PsbP channel in green plant PSII. The 137-140 polyG-reconstituted PSII, however, recovered nearly 70% of the oxygen-evolving activity of WT-reconstituted PSII. This is likely due to the flexibility of the loop consisting of six glycines in a row, including residues PsbP-Gly141 and Gly142.

Surprisingly, several mutations at Asp139 increased the oxygen-evolving activity of PSII. The enhancement was especially pronounced with the D139N mutation. PsbP is known to play a critical role in retaining Cl^−^, but at low Cl^−^ concentrations, PsbQ is additionally required for optimal oxygen-evolving activity (58, 79). However, while the oxygen-evolving activity under extremely low Cl^−^ concentrations (without PsbQ) was low with WT-reconstituted PSII (70% relative to activity with sufficient Cl^−^), D139N-reconstituted PSII showed a high activity of around 95% relative to the activity of WT-reconstituted PSII with sufficient Cl^−^ (Fig. 4). In addition, the D139N mutation had little effect on the binding ability of PsbP to PSII (Fig.3B). These results clearly suggest that the D139N mutation strengthened the Cl^−^ retaining ability of PsbP. The site of mutation is in proximity to Cl-2 rather than Cl-1; PsbP-Loop 4 almost directly interacts with the Cl-2 binding site, whereas the Cl-1 binding site is set apart (Fig. 1B). In addition, theoretical calculations suggested that Cl-2 is weakly bound to PSII compared to Cl-1 (17). Although the possibility of this mutation having a distal effect on the Cl-1 binding site cannot be excluded, there would be more pronounced effects on the Cl-2 binding site. Therefore, it can be predicted that Cl-2 is the Cl^−^ whose binding was especially stabilized by the mutation. While only the importance of Cl-1 has been revealed to date (36), the above observations suggest that not only Cl-1 but also Cl-2 can be important for the oxygen-evolving activity of PSII.

As mentioned earlier, the Cl-2 binding site comprises the backbone groups of D1-Asn338, D1-Phe339, and CP43-Glu354 (2). Therefore, it can be expected that if the binding of Cl-2 was stabilized by the PsbP-D139N mutation, this mutation may have affected the protein structure to induce movement of the polypeptide backbones near Cl-2. The results of FTIR analyses indicate that this mutation induced a unique structural change involving movement of the polypeptide backbone at a Cl^−^ binding site (Fig. 6). The present theoretical calculations showed that the PsbP-D139N mutation leads to hydrogen bond formation between the PsbP-Asn139 side chain and the backbone carboxyl group of D2-Leu353 (Fig. 7). As molecular dynamics simulation indicated that Cl-2 is likely to be incorporated into the binding cavity via D2-Leu353 (18), the PsbP-D139N mutation can affect the Cl-2 binding. Several other mutations at PsbP-D139, such as D139A, E, F, and Q, slightly increased the oxygen-evolving activity of PSII; however, they are much less effective than the D139N mutation and may induce other structural changes.

The side chains of D1-Asn338 and CP43-Glu354, whose backbone groups serve as the binding sites of Cl-2, are involved in the proton-conducting hydrogen-bonded network of the O4-water chain (25, 26, 80), fixing the positions of the water molecules and preorganizing the hydrogen-bond pattern of the water molecules for proton transfer (81). Thus, Cl-2 may contribute to the formation of the ordered water chain, which is a prerequisite for efficient proton transfer (82). In particular, the water molecule (W538) at the CP43-Glu354 moiety in the O4-water chain is exchangeable with water molecules in the bulk region (18). Thus, it seems possible that changes at the Cl-2 binding moiety caused by the PsbP-D139N mutation affects not only the proton transfer along the O4-water chain but also the uptake of exchangeable water molecules at the Mn_4_CaO_5_ moiety.

**Fig. 8.**
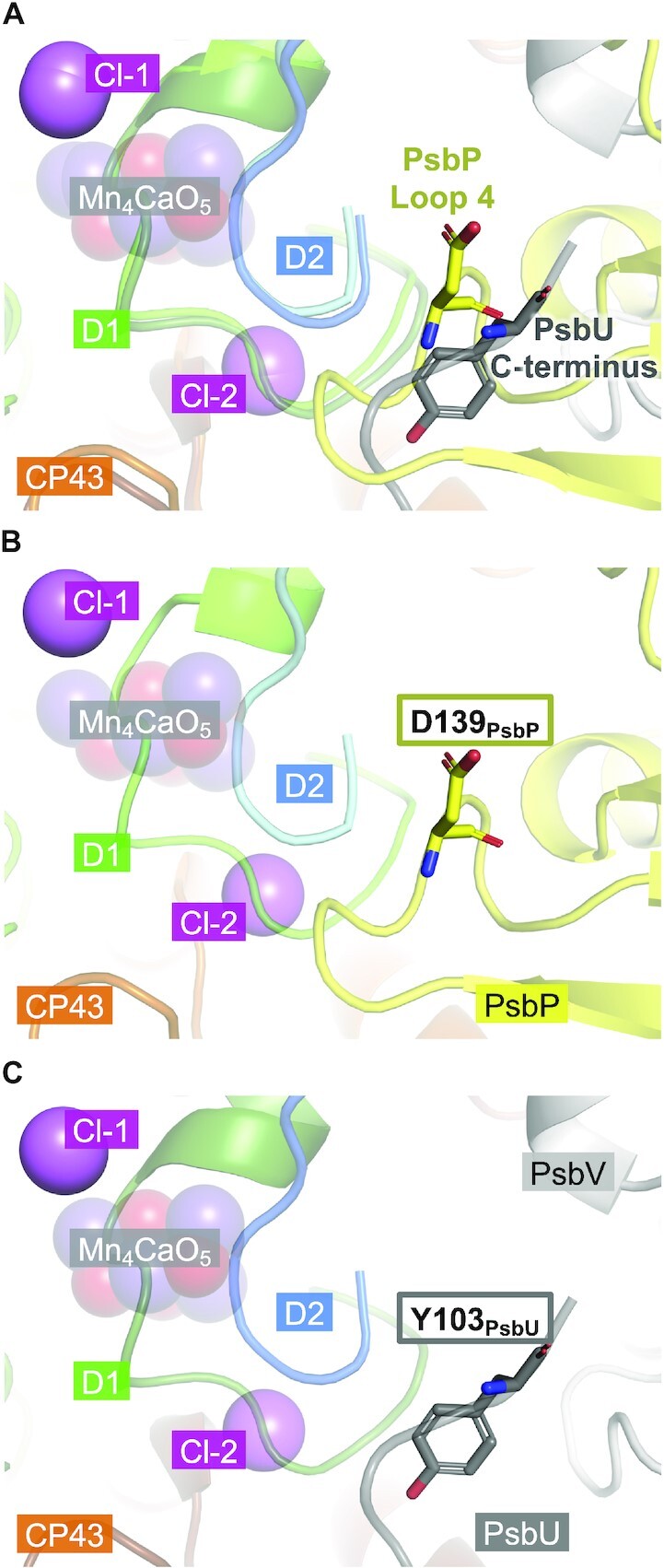
Comparison of the structure of cyanobacterial PSII and green plant PSII. (A) Superposition of (B) the structure of green plant PSII (PDB ID: 5XNL) surrounding PsbP-Loop 4 and (C) the structure of cyanobacterial PSII (PDB ID: 3WU2) surrounding the C-terminus of PsbU. D1, D2, CP43, PsbP (in A and B), PsbU (in A and C), and PsbV (in A and C) are shown in green, blue, orange, yellow, dark gray, and light gray, respectively. The Cl^−^ are shown as pink spheres, and residues PsbP-Asp139 and PsbU-Tyr103 are shown as stick models. The glycerol molecules of the cyanobacterial PSII have been removed for clarity.

Comparing the structures of green plant PSII (7) and cyanobacterial PSII (2, 3), the Loop 4 region of PsbP in green plant PSII was found to have spatially replaced the C-terminal region of PsbU in cyanobacterial PSII (36) (Fig. 8). In the location corresponding to PsbP-Asp139 of green plant PSII, PsbU-Tyr103 of cyanobacterial PSII was observed. This tyrosine residue of PsbU is completely conserved among cyanobacterial PSII, red algal PSII, and diatom PSII, and its functional importance has been reported in studies using red algal PSII (83–85). These studies illustrated that the aromatic ring of PsbU-Tyr92 in red algal PSII (corresponding to PsbU-Tyr103 in cyanobacterial PSII) is important for optimizing the availability of Cl^−^ for water oxidation. Their results showed that while lack of only the C-terminal residue (Lys93) of PsbU had a minor effect on the oxygen-evolving activity of PSII, the lack of two residues (Lys93 and Tyr92) from the C-terminus resulted in a remarkable decrease in activity in the absence of Cl^−^ and Ca^2+^, which could be significantly recovered by addition of NaCl, and even more so by addition of CaCl_2_. Similar results were observed with the substitution of PsbU-Tyr92, except for the PsbU-Y92F mutation, which almost fully recovered the function of PsbU, and the PsbU-Y92H mutation, which partially recovered it. They concluded that this conserved tyrosine residue of PsbU would interact with D1-Pro340 located in the C-terminal loop region of D1, and that this interaction, which would affect the structure of the C-terminal loop region of D1, was important for the optimal function of the Mn_4_CaO_5_ cluster. With the knowledge that Cl-2 binds to PSII surrounded by the C-terminal loop region of D1, it can be predicted that the van der Waals contact between D1-Pro340 and this conserved tyrosine residue of PsbU plays a crucial role especially in stabilizing the binding of Cl-2 to PSII in red algae, and most likely also in cyanobacteria and diatoms. Our results show that the Loop 4 region of PsbP is important for the oxygen-evolving activity of green plant PSII, and that it could be involved in the retention of Cl-2. This suggests that PsbP-Loop 4 replaces the C-terminal region of PsbU functionally as well as spatially.

The Cl^−^ concentration in the thylakoid lumen has not been determined; however, it has been predicted to be in the range of approximately 5.5 to 86 mM, based on the assumption that all of the chloroplast Cl^−^ is in the thylakoid lumen (86). The actual concentration can be lower, as the assumption contradicts with the findings that the Cl^−^ ion flux across the thylakoid lumen regulates the electrochemical proton gradient, also known as the proton motive force (PMF) (87, 88). Recent studies on thylakoid-located Cl^−^ channels show that the Cl^−^ ion flux, linked to partitioning of the electrical (Δ*ψ*) and chemical (ΔpH) components of the PMF, is affected by the light-condition, and contributes to the fine-tuning of photosynthetic electron transport and photoprotection (89–93). From these studies, the Cl^−^ concentration in the lumen is likely to be lower under weaker light intensities, and further lower in the dark. In a different study using cyanobacteria (*Synechocystis* sp. PCC 6803), a *psbV*-deficient mutant, which requires a high Cl^−^ concentration due to inefficient Cl^−^ retention in PSII, was incubated in Cl^−^-free medium in order to isolate spontaneous suppressor mutants that do not require external Cl^−^ (94). A total of three independent suppressor mutations were found, and they were all located at the *slr0753* gene, which they considered a putative chloride efflux transporter gene. This also suggests that the Cl^−^ concentration at the lumenal side of PSII can be lower than expected, and indicates the physiological importance of supporting Cl^−^ retention in the OEC.

In green plant PSII, PsbQ is involved in the retention of Cl^−^ under low Cl^−^ conditions: especially below 10 mM Cl^−^ (95). However, the FTIR analysis suggests that the PsbP-D139N mutation leads to a unique structural change in the OEC, which differs from that in PSII binding both WT-PsbP and PsbQ (Fig. 6). Furthermore, even in the presence of PsbQ under Cl^−^ sufficient conditions, the oxygen-evolving activity of D139N-reconstituted PSII tends to be higher than that of WT-reconstituted PSII (Figure S6, Supplementary Material). In contrast to that of WT-reconstituted PSII, the oxygen-evolving activity of D139N-reconstituted PSII is only slightly affected by the addition of PsbQ. These data suggest that the mechanisms by which Cl^−^ retention is enhanced, differs between that by PsbQ and that by D139N-PsbP.

It has been suggested that the Cl^−^ binding is not only essential for the oxygen-evolving activity, but is also required for preventing the formation of reactive oxygen species (96–100). Then, the question remains as to why plants have not selected PsbP-Asn139 instead of PsbP-Asp139 during evolution. The amino acid sequence of PsbP-Loop 4 is widely conserved from dicots to green algae, and mutations at Asp139 were only found in species possessing multiple PsbP isoforms, including at least one isoform that does not have a mutation at this site (Figure S1, Supplementary Material). It is possible that PsbP-Asp139 has an unknown role in vivo. Another possibility may be that moderate Cl^−^ retention at the Cl-2 binding site due to PsbP-Asp139 is preferable in vivo, for example, for regulation of PSII activity. Further in vivo analyses are required to reveal why PsbP-Asp139 is conserved among green plant species.

## Materials and methods

### Preparation of recombinant proteins

The coding region for the mature PsbP protein of *Spinacia oleracea* was inserted into expression vectors pET-41a (Novagen) and pET-21d (Novagen). Using a site-directed mutagenesis kit (Agilent), expression plasmids for the mutated PsbP proteins Δ137–140, D137N, D139A, D139E, D139F, D139K, D139N, D139Q, and E140Q were constructed. A tag consisting of six histidine (6xHis) residues was attached to the C-terminus of the WT and mutated PsbP by inverse PCR, and the expression plasmid for the His-tagged mutated PsbP protein, 137-140 polyG-6xHis, was constructed by inverse PCR with the expression plasmid for WT-6xHis as template. After transformation of *Escherichiacoli* cells (strain BL21 (DE3)) with the resulting constructs, expression of recombinant WT (-6xHis) and mutated PsbP (-6xHis) proteins was performed based on the procedure reported previously (101). To purify recombinant PsbP proteins without His-tags, the *E. coli* cells were sonicated in buffer P (10 mM MES-NaOH, 40 mM NaCl, pH 5.5), and the soluble fraction of the resulting bacterial homogenate was dialyzed with buffer P as dialysis buffer. After centrifugation, the supernatant was applied to an ion exchanger, CM Sepharose CL-6B (Cytiva), and the recombinant PsbP proteins were eluted by a linear gradient from 40 mM NaCl up to 1 M NaCl. As for purification of recombinant PsbP-6xHis proteins, the *E. coli* cells were sonicated in buffer A (0.1 M sodium phosphate buffer (Na-PB), 0.3 M NaCl, pH 7.8 to 8.0) with protease inhibitor (cOmplete, EDTA-free Protease Inhibitor Cocktail, MilliporeSigma) added. The soluble fraction of the resulting bacterial homogenate was applied to cOmplete His-Tag Purification Resin (Roche) pre-equilibrated with buffer A, and recombinant PsbP-6xHis proteins were eluted by a stepwise gradient of up to 250 mM imidazole in buffer A. Finally, NaCl and imidazole was removed by dialysis. Recombinant PsbP proteins without His-tags were only used in light-induced FTIR analysis; elsewhere, recombinant PsbP-6xHis proteins were used. Preparation of the recombinant PsbQ protein was performed as reported by Kakiuchi et al. (66), and NaCl was removed from the purified PsbQ protein by a combination of ultrafiltration and the use of PD-10 desalting columns (Cytiva).

### Reconstitution experiments

Oxygen-evolving PSII membranes (102) were isolated from market spinach based on previously reported methods (103). The initial oxygen-evolving activity of the PSII membranes under 0.2 mM Cl^−^ and 5 mM CaCl_2_ were 332 to 361 µmol O_2_ mg Chl^−1^ h^−1^ and 385 to 398 µmol O_2_ mg Chl^−1^ h^−1^, respectively. Reconstitution of recombinant PsbP proteins to NaCl-washed PSII membranes was performed as described by Seidler (104) with partial modification. PSII membranes (0.1 mg Chl) were incubated in 1 mL NaCl buffer (25 mM MES-NaOH (pH 6.5), 1.5 M NaCl, 0.4 M sucrose) on ice for 30 min and were then washed once with NaCl buffer containing 50 µM EGTA. Reconstitution was carried out in reconstitution buffer (25 mM MES-NaOH (pH 6.5), 20 mM CaCl_2_, 0.4 M sucrose) with a molar ratio of 4 PsbP proteins (and 4 PsbQ proteins where indicated) per PSII reaction center unless indicated. PSII levels were determined with the assumption of 200 chlorophylls per PSII reaction center, and the protein concentrations for PsbP (23 kDa) and PsbQ (18 kDa) were determined by Bradford assay using BSA standards. After reconstitution, the 20 mM CaCl_2_ and unbound PsbP (and PsbQ) proteins were removed by washing with the buffer. Both NaCl treatment and reconstitution were performed in the dark. Using a Clark-type oxygen electrode (Hansatech, UK) at 25°C, oxygen evolution of PSII membranes (10 µg Chl mL^−1^) was measured in activity measurement buffer (25 mM MES-NaOH (pH 6.5, unless indicated), 0.4 M sucrose) with 0.4 mM DCBQ as electron acceptor under saturating light illumination (2,500 µmol photons m^−2^ s^−1^). When measuring the oxygen-evolving activity under various Cl^−^ concentrations, NaCl was added to the activity measurement buffer. Binding of PsbP to PSII was confirmed by SDS-PAGE using 12.5% acrylamide gels containing 6 M urea. Gels were stained with Oriole (Bio-Rad), bands were visualized using a gel imaging instrument (ChemiDoc Touch Imaging System; Bio-Rad), and the relative amount of PsbP bound to PSII was determined using Image Lab Software, Version 5.2.1 (Bio-Rad). Each reconstitution experiment was conducted at least twice to confirm the reproducibility of the results.

### FTIR measurements

Sample preparation and FTIR measurements were performed using methods (60, 76) based on previous reports (59, 66, 75). The Cl^−^ concentration of the sample during the assay was 15 mM.

### Theoretical calculations

As a basis for the computations, a cryo-EM structure of plant PSII (PDB ID: 5XNL) (7) was used. The PsbP-D139N mutant PSII structure was modeled by replacing the sidechain of aspartate with that of asparagine. Hydrogen atoms and missing heavy atoms in the cryo-EM structure were generated, and the entire geometries were energetically optimized using the all-atom CHARMM22 parameter set (105) with CHARMM version 40b (106). The energy minimization was performed with the steepest descents algorithm followed by the conjugate gradients algorithm (106). During the process, all atoms in D1, D2, CP43, PsbP, and PsbQ subunits, CP47-Arg384, and the generated atoms were relaxed; all titratable groups were kept in their standard protonation states (i.e. acidic and basic groups were ionized). The atomic charges of the cofactors were taken from our previous studies on PSII (107). The dielectric constant of 4 was used for the protein interior. For atomic coordinates, see Dataset S1 (Supplementary Material).

## Supplementary Material

pgac136_Supplemental_Files

## Data Availability

All data are presented within the manuscript or are available in the Supplementary Materials.
