# Supplementary material for: D139N mutation of PsbP enhances the oxygen-evolving activity of photosystem II through stabilized binding of a chloride ion
Source: PNAS Nexus. 2022 Jul 23;1(3):pgac136. doi: 10.1093/pnasnexus/pgac136 (PMC9896922; doi:10.1093/pnasnexus/pgac136)
Supplement: pgac136_Supplemental_Files [file pgac136_supplemental_files.zip › PNASNEXUS-PNASNEXUS-2021-00266-T-s01.pdf]

PNAS nexus

Supplementary Information for
D139N mutation of PsbP enhanced the oxygen-evolving activity of photosystem II through stabilized binding of a chloride ion

Ko Imaizumi, Taishi Nishimura, Ryo Nagao, Keisuke Saito, Takeshi Nakano, Hiroshi Ishikita, Takumi Noguchi, and Kentaro Ifuku

Corresponding author: Kentaro Ifuku
Email: ifuku.kentaro.2m@kyoto-u.ac.jp

This PDF file includes:

Figures S1 to S6
Tables S1 to S3

SI References

Other supplementary materials for this manuscript include the following:

Datasets S1

```
                                                    *:    :* *:.** *** *: : *::
Spinacia oleracea                                   YYSITVLTRTADGDEGGKHQVIAATV
Arabidopsis thaliana (PsbP-1)                       YYYLSVLTRTADGDEGGKHQLITATV
Eutrema salsugineum (a)                             YYYLSVLTRTADGDEGGKHQLITATV
Eutrema salsugineum (b)                             YYYLSVLTRTADGDEGGKHQLITATV
Eutrema salsugineum (c)                             YYYLSVLTRTADGDEGGKHQLITATV
Nicotiana tabacum (1A)                              YYILSVLTRTADGDEGGKHQLISATV
Nicotiana tabacum (2AF)                             YYYLSILTRTADGNEGGKHQLVTATV
Nicotiana tabacum (3F)                              YYYLSVLTRTADGNEGGKHQLITATV
Nicotiana tabacum (5B)                              YYILSVLTRTADGDEGGKHQLISATV
Pisum sativum (a)                                   YYNISVLTRTADGDEGGKHQLITATV
Pisum sativum (b)                                   YYVLSVLTRTADGDEGGKHQIIRATV
Populus alba (a)                                    YYFLSVLTRTADGDEGGKHQLITATV
Populus alba (b)                                    YFFLSVLTRTADGDEGGKHQLITATV
Ananas comosus (a)                                  YYFLSVLTRTADGDEGGKHQLITATV
Ananas comosus (b)                                  YYYISVLTRTADGDEGGKHHLITATV
Ananas comosus (c)                                  YYYISVLTRTADGDEGGKHHLITATV
Oryza sativa Japonica Group                         YYSVTVLTRTADGDEGGKHQLITATV
Triticum aestivum                                   YYSITVLTRTADGDEGGKHQLITATV
Zea mays (a)                                        YYAVSVLTRTADGDEGGKHQLIAATV
Zea mays (b)                                        YYSITVLTRTADGDEGGKHQLITATV
Selaginella moellendorffii (a)                      YYDLDVLTRTADGDEGGTHQLIVASI
Selaginella moellendorffii (b)                      YYDLDVLTRTADGDEGGTHQLIVASI
Selaginella moellendorffii (c)                      YYDLDVLTRTADGDEGGTHQLIVASI
Marchantia polymorpha subsp. ruderalis (a)          YFYISVLTRTADGDEGGKHQLIIATI
Marchantia polymorpha subsp. ruderalis (b)          YYNLEVLTRTGDGNEGGRHQCIVATV
Marchantia polymorpha subsp. ruderalis (c)          YYYLEVLTRTGDGTEGGRHQCIVATV
Chlamydomonas reinhardtii                           YYKYELLVRSADGDEGGRHQLIGATV
Coccomyxa subellipsoidea C-169 (a)                  YYKYEILTRTADGDEGGRHQLITAAV
Coccomyxa subellipsoidea C-169 (b)                  YYKYDILSRSADGNEGGKHNLITAAV
Bigelowiella natans                                 YYNYELLSRTADGDEGGRHKLVSAAV
```

Fig. S1. Amino acid sequence alignment of the region including PsbP-Loop 4. The box indicates the four residues from Asp137 to Glu140 located in PsbP-Loop 4. Conserved residues are shown on gray background. Each PsbP amino acid sequence of the species with several *psb*P genes is identified with an alphabet in parenthesis, except for those of *Nicotiana tabacum* and *Arabidopsis thaliana*. PsbP-2 of *Arabidopsis thaliana* is not included, since it has been reported not to accumulate in the ecotype Columbia-0 (Col-0) due to a frameshift (1, 2). The accession numbers of the used *psb*P sequences are shown in Table S1. Note that some of these *psb*P sequences are that of hypothetical or uncharacterized proteins.

Table S1. Accession numbers of the *psb*P sequences used in the amino acid sequence alignment (Fig. S1). The sequences were obtained from NCBI (National Center for Biotechnology Information) except for the two sequences of *Pisum sativum*, which were obtained from URGI (Unité de Recherches en Génomique Info).

**Sequence name**	**Accession number**
Spinacia oleracea	XP_021843617.1
Arabidopsis thaliana (PsbP-1)	NP_172153.1
Eutrema salsugineum (a)	XP_006410179.1
Eutrema salsugineum (b)	XP_006417894.1
Eutrema salsugineum (c)	XP_024008923.1
Nicotiana tabacum (1A)	CAA41712.1
Nicotiana tabacum (2AF)	CAA44293.1
Nicotiana tabacum (3F)	CAA39039.1
Nicotiana tabacum (5B)	CAA44292.1
Pisum sativum (a)	Psat5g189160.1 (URGI BLAST)
Pisum sativum (b)	Psat2g018280.1 (URGI BLAST)
Populus alba (a)	XP_034915063.1
Populus alba (b)	XP_034931158.1
Ananas comosus (a)	XP_020096384.1
Ananas comosus (b)	XP_020080815.1
Ananas comosus (c)	XP_020087457.1
Oryza sativa Japonica Group	XP_015646728.1
Triticum aestivum	AYI50055.1
Zea mays (a)	XP_008652004.2
Zea mays (b)	NP_001310827.1
Selaginella moellendorffii (a)	XP_002991817.2
Selaginella moellendorffii (b)	XP_002992976.2
Selaginella moellendorffii (c)	XP_024521362.1
Marchantia polymorpha subsp. *ruderalis* (a)	OAE26568.1
Marchantia polymorpha subsp. *ruderalis* (b)	OAE29654.1
Marchantia polymorpha subsp. *ruderalis* (c)	OAE29655.1
Chlamydomonas reinhardtii	XP_001694126.2
Coccomyxa subellipsoidea C-169 (a)	XP_005649549.1
Coccomyxa subellipsoidea C-169 (b)	XP_005649550.1
Bigelowiella natans	AAP79210.1

Table S2. Oxygen-evolving activity of PSII reconstituted with various PsbP mutant proteins (summarized in Fig. 2*A*). The values shown are mean ± S.D. (n = 3, technical replicates), and asterisks (*) following the type of PsbP indicate statistical differences based on Dunnett's test with WT as control (*P < 0.05, **P < 0.01, ***P < 0.001).

PsbP	Oxygen-evolving activity (μmol O_2 mg Chl^{-1} h^{-1})
WT	177 ± 8
−PsbP ***	16 ± 1
Δ137-140 ***	53 ± 12
D137N ***	46 ± 2
137-140 polyG ***	125 ± 10
WT	190 ± 4
D139A *	249 ± 28
D139F *	244 ± 21
D139N ***	297 ± 33
D139Q	208 ± 3
WT	179 ± 6
D139E *	210 ± 16
D139K **	139 ± 7
E140Q **	137 ± 12

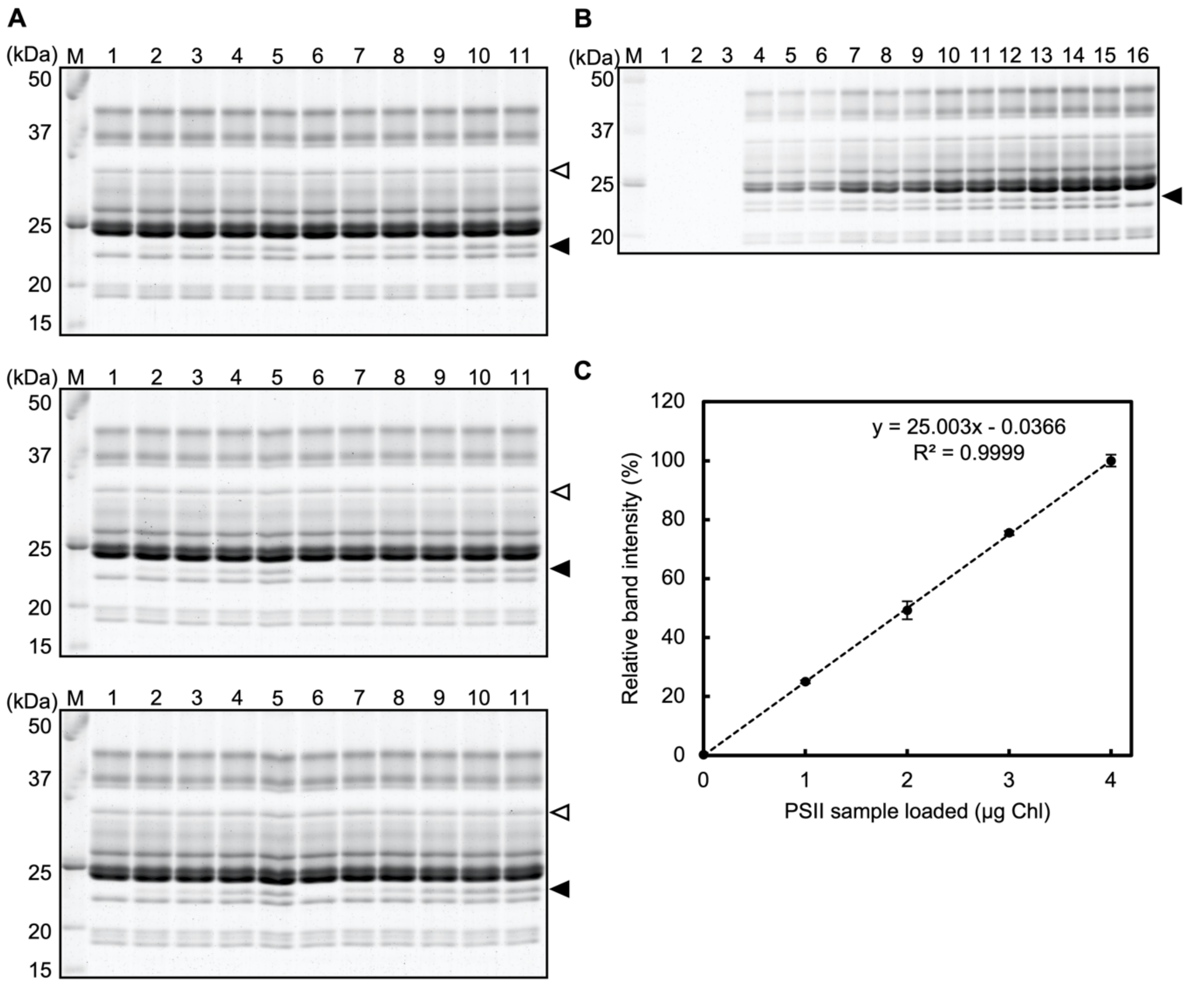

Fig. S2. Quantification of PsbP-6xHis bands of Oriole (Bio-Rad) stained SDS-PAGE gels. (A) The SDS-PAGE gels used for determining the extent of PsbP binding in Fig. 3*B*. The amount of PSII samples loaded onto the gel was normalized based on the PsbO band intensity. The PsbO bands and PsbP-6xHis bands are indicated with open and filled arrowheads, respectively. Lane M: Precision Plus Protein Unstained Standards (Bio-Rad); lanes 1, 2, 3, 4, and 5: WT-reconstituted PSII with a reconstitution step with 0, 0.5, 1, 2, and 4 PsbP proteins per PSII reaction center, respectively; lanes 6, 7, 8, 9, and 10: D139N-reconstituted PSII with a reconstitution step of 0, 0.5, 1, 2, and 4 PsbP proteins per PSII reaction center, respectively; lane 11: WT-reconstituted PSII with reconstitution at a ratio of PsbP:PSII = 4:1 (control for D139N-reconstituted PSII). (B and C) The accuracy of determination of the levels of PsbP binding. (B) Serial dilutions (0, 1, 2, 3, and 4 µg Chl) of WT-reconstituted PSII were run on an SDS-PAGE gel, and the gel was Oriole stained. The PsbP-6xHis bands are indicated with an arrowhead. Lane M: Precision Plus Protein Unstained Standards (Bio-Rad); lanes 1–15: 0 µg Chl (lanes 1–3), 1 µg Chl (lanes 4–6), 2 µg Chl (lanes 7–9), 3 µg Chl (lanes 10–12), and 4 µg Chl (lanes 13–15) of WT-reconstituted PSII; lane 16: −PsbP (NaCl-washed PSII without reconstitution of PsbP). (C) Relative PsbP-6xHis band intensities of the Oriole stained SDS-PAGE gel. The mean PsbP-6xHis band intensity of the three lanes (lanes 13–15 of Fig. S2*B*) where 4 µg Chl of PSII was loaded onto was set as 100%; each data point shows the mean PsbP-6xHis band intensity from each set of three lanes, error bars = S.D.

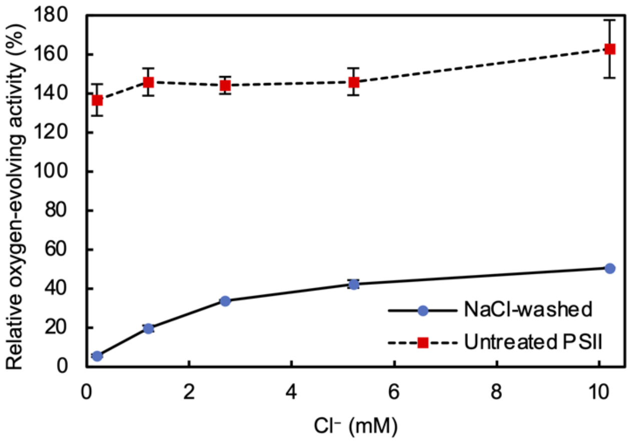

Fig. S3. Cl^- dependence of the oxygen-evolving activity of untreated and NaCl-washed PSII. The oxygen-evolving activity of untreated (blue circle) and NaCl-washed (red square) PSII was measured in the presence of various concentrations of Cl^- at pH 6.5. Oxygen-evolving activity of WT-reconstituted PSII under 10.2 mM Cl^- (247–260 μmol O_2 mg Chl^{-1} h^{-1} in independent experiments) was set as 100%; error bars = S.D. (*n* = 3, technical replicates).

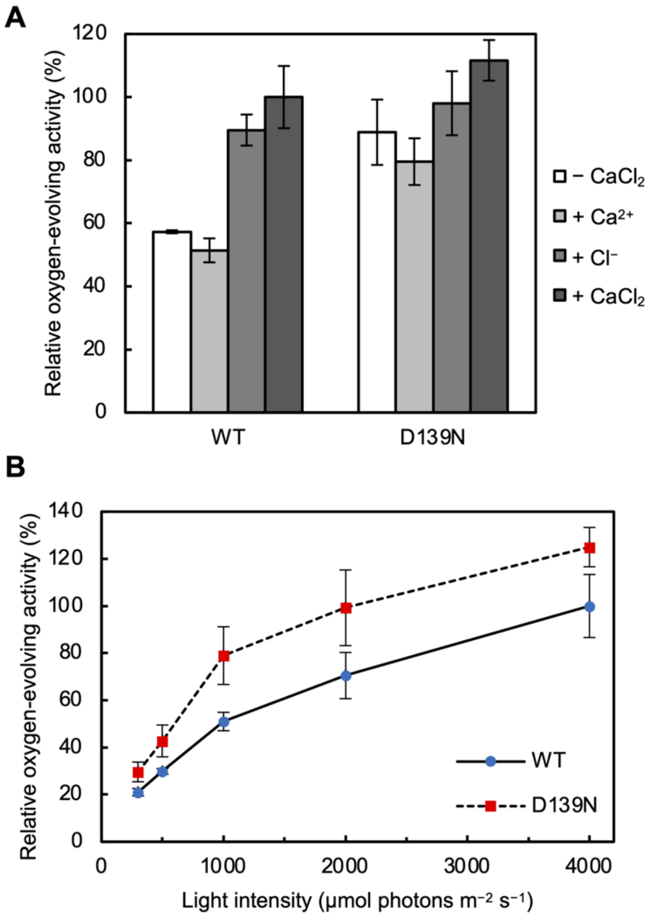

Fig. S4. Effects of ions and light intensities on the oxygen-evolving activity of WT- and D139N-reconstituted PSII. (A) Ca^{2+} and Cl^{-} dependence of the oxygen-evolving activity of WT- and D139N-reconstituted PSII. The different ion conditions were as follows: no addition of Ca^{2+} or Cl^{-} (*white bars*), 5 mM Ca^{2+} (*light gray bars*), 10 mM Cl^{-} (*gray bars*), and 5 mM $CaCl_2$ (*dark gray bars*). All conditions contained an additional 0.2 mM Cl^{-}. Oxygen-evolving activity of WT-reconstituted PSII under 5 mM $CaCl_2$ (279 μmol O_2 mg Chl^{-1} h^{-1}) was set as 100%; error bars = S.D. (*n* = 3, technical replicates). (B) Light saturation curves of the oxygen-evolving activity of WT- (blue circles) and D139N- (red squares) reconstituted PSII. Oxygen-evolving activity of WT-reconstituted PSII under 4000 μmol photons m^{-2} s^{-1} (161 μmol O_2 mg Chl^{-1} h^{-1}) was set as 100%; error bars = S.D. (*n* = 3, technical replicates).

Table S3. Oxygen-evolving activity of WT- and D139N-reconstituted PSII in the presence of different artificial electron acceptors. Oxygen-evolving activity under 0.2 mM Cl^- was measured in the presence of 0.4 mM DCBQ (2,6-dichloro-*p*-benzoquinone), DMBQ (2,6-dimethyl-*p*-benzoquinone), or PPBQ (phenyl-*p*-benzoquinone). The values shown are mean ± S.D. (n = 3, technical replicates), and for the relative oxygen-evolving activity, the oxygen-evolving activity of WT-reconstituted PSII with each electron acceptor was set as 100%.

Artificial electron acceptors	PsbP	Oxygen-evolving activity (μmol O_2 mg Chl^{-1} h^{-1})	Relative oxygen-evolving activity (%)
DCBQ	WT	161 ± 3	100 ± 2
	D139N	220 ± 10	137 ± 6
DMBQ	WT	108 ± 6	100 ± 5
	D139N	153 ± 12	141 ± 11
PPBQ	WT	279 ± 28	100 ± 10
	D139N	372 ± 6	133 ± 2

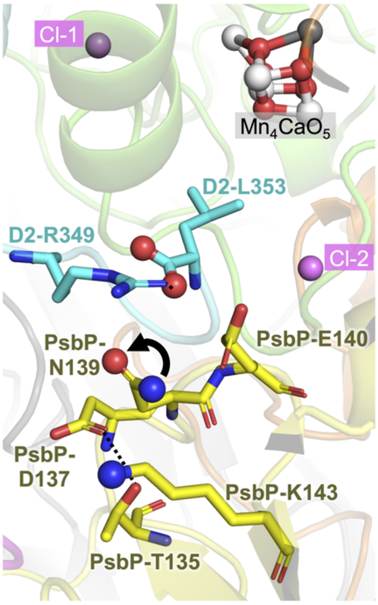

Fig. S5. Alternative conformation of the PsbP-D139N mutant. The Nδ atom of PsbP-Asn139 is oriented to the side chain of PsbP-Lys143. The calculated energy was 16.1 kcal/mol higher than that of the original conformation shown in Fig. 7*B*.

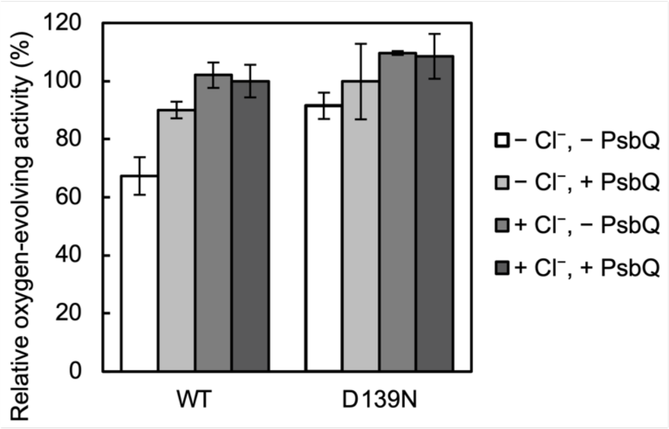

Fig. S6. Effects of Cl^- and PsbQ on the oxygen-evolving activity of WT- and D139N-reconstituted PSII. The different conditions were as follows: 0.2 mM Cl^- in the absence of PsbQ (*white bars*), 0.2 mM Cl^- in the presence of PsbQ (*light gray bars*), 10.2 mM Cl^- in the absence of PsbQ (*gray bars*), and 10.2 mM Cl^- in the presence of PsbQ (*dark gray bars*). For the "+PsbQ" conditions, the reconstitution experiments were performed with 4 PsbQ proteins together with 4 PsbP proteins per PSII reaction center during the reconstitution step. Oxygen-evolving activity of WT-reconstituted PSII under 10.2 mM Cl^- in the presence of PsbQ (261 μmol O_2 mg Chl^{-1} h^{-1}) was set as 100%; error bars = S.D. (*n* = 3, technical replicates).

Dataset S1: The atomic coordinate used to create Fig. 7.

SI References

1. S. Ishihara, *et al.*, Distinct Functions for the Two PsbP-Like Proteins PPL1 and PPL2 in the Chloroplast Thylakoid Lumen of Arabidopsis. *Plant Physiol.* **145**, 668–679 (2007).
2. K. Ifuku, S. Ishihara, R. Shimamoto, K. Ido, F. Sato, Structure, function, and evolution of the PsbP protein family in higher plants. *Photosynth. Res.* **98**, 427–437 (2008).
